# Quality of Life after post-prostatectomy intensity modulated radiation therapy to the prostate bed with or without the use of gold fiducial markers for image guidance or higher total radiotherapy doses

**DOI:** 10.1590/S1677-5538.IBJU.2016.0189

**Published:** 2017

**Authors:** Yazan A. Abuodeh, Arash O. Naghavi, Tzu-Hua Juan, Zhenjun Ma, Richard B. Wilder

**Affiliations:** 1 Department of Radiation Oncology, Moffitt Cancer Center, Tampa, Florida, USA;; 2 Cancer Treatment Centers of America, Newnan, Georgia, USA

**Keywords:** Prostate, Postoperative Care, Radiotherapy, Quality of Life

## Abstract

**Purpose:**

To evaluate quality of life (QoL) after post-prostatectomy intensity modulated radiation therapy (IMRT) in the “adjuvant” setting starting within 4 months of radical prostatectomy for adverse features; and “salvage” setting for a PSA≥0.2ng/mL.

**Materials and Methods:**

Retrospective review of 130 patients who underwent IMRT to the prostate bed±gold fiducial marker placement for image guidance to 64.8-72.0Gy (median, 70.2Gy) between 2004 and 2013. Higher doses were defined as 70.2-72.0Gy and lower doses were defined as 64.8-68.4Gy. Androgen deprivation therapy (ADT) was given to 4/48 (8%) adjuvant patients and 9/82 (11%) salvage patients. International Prostate Symptom Score (IPSS), Sexual Health Inventory for Men (SHIM), and Expanded Prostate Cancer Index Composite-26-bowel (EPIC-26-bowel) questionnaires were used to assess urinary, sexual, and bowel QoL, respectively.

**Results:**

Median follow-up was 46 months. There were better urinary (p=0.03) and sexual (p=0.002) QoL scores with adjuvant IMRT relative to salvage IMRT. The use of prostate bed fiducial markers did not significantly affect urinary, sexual, or bowel QoL (p=0.39, p=0.49, and p=0.40, respectively). Higher total radiotherapy doses did not significantly affect urinary, sexual, or bowel QoL (p=0.21, p=0.61, and p=0.36, respectively).

**Conclusions:**

There was no significant change in urinary, sexual, and bowel sexual QoL with post-prostatectomy IMRT regardless of whether prostate bed fiducial markers or higher total radiotherapy doses were used. QoL with IMRT in the present study compares favorably with prior reports for three-dimensional conformal radiation therapy.

## INTRODUCTION

Three Phase III studies have shown a benefit to post-prostatectomy radiation therapy (1-4). There is no consensus on the definitions of post-prostatectomy “adjuvant” radiotherapy versus “salvage” radiotherapy. Similar to the Tasman Radiation Oncology Group Radiotherapy and Androgen Deprivation In Combination After Local Surgery (RAVES) trial (5, 6), this study defines adjuvant radiotherapy as treatment starting within 4 months of radical prostatectomy for positive surgical margins, extraprostatic extension, or seminal vesicle invasion and salvage radiotherapy as treatment for a post-prostatectomy prostate-specific antigen (PSA) measurement≥0.2ng/mL.

Physicians under-estimate worsening and over-estimate improvement in symptoms relative to patients (7). As a result, it is important to measure patient-reported outcomes when assessing quality of life (QoL). The primary aim of this retrospective observational study is to assess urinary, sexual, and bowel QoL prior to and following the delivery of post-prostatectomy intensity modulated radiation therapy (IMRT) to the prostate bed. QoL is compared in patients who received adjuvant IMRT versus salvage IMRT. Also, QoL is examined in patients who did or did not undergo placement of gold fiducial markers in their prostate bed for image guidance or received higher versus lower total radiotherapy doses.

## MATERIALS AND METHODS

### Patient Characteristics

After obtaining institutional review board approval, the authors reviewed the medical records of 130 prostatectomy patients who did not have evidence of regional or distant metastases on computed tomography scans of the abdomen and pelvis and bone scans prior to the initiation of IMRT. Patients underwent radiotherapy at a single institution between 2004 and 2013. Patients were included in this study if they underwent IMRT to the prostate bed alone. Patients were excluded if they underwent elective pelvic lymph node irradiation since it can result in worse acute urinary and bowel QoL (8). Twenty-eight percent of radical prostatectomy patients had positive surgical margins, extraprostatic extension, or seminal vesicle invasion and were referred within one month of surgery for consultation regarding the option of adjuvant radiotherapy beginning within 4 months of surgery.

### Urinary, Sexual, and Bowel Quality of Life Questionnaires

When QoL data started to be collected in 2004 at our center, the International Prostate Symptom Score (IPSS) questionnaire was a popular method of assessing urinary QoL (9). As a result, this survey was adopted by the Department of Radiation Oncology and Department of Urology to assess urinary QoL. IPSS scores range from 0 to 35 with lower scores indicating a higher urinary QoL. Similarly, the SHIM questionnaire was commonly used in 2004 to assess sexual QoL (8). Consequently, this survey was adopted by the Department of Radiation Oncology and Department of Urology to grade sexual QoL and used in this report. SHIM scores range from 1 to 25 with higher scores indicating better sexual QoL. The Expanded Prostate Cancer Index Composite-26 (EPIC-26) instrument is a research tool used for capturing patient-reported QoL outcomes related to the domains of bladder, sexual, and bowel functioning for men undergoing treatment for prostate cancer (10). In this study, EPIC-26 was used to assess bowel (EPIC-26-bowel) QoL. The bowel summary score can range from 0 to 100, with a higher score indicating a better QoL. The urinary and sexual domains of the EPIC-26 form were not given to patients because IPSS and Sexual Health Inventory for Men (SHIM) forms were used to assess urinary and sexual QoL, respectively. In 2015, the International Consortium for Health Outcomes Measurement recommended EPIC-26 as the preferred method for measuring QoL in men with localized prostate cancer (11). As a result, we presently use EPIC-26 to determine urinary, sexual, and bowel QoL. The IPSS, SHIM, and EPIC-26 questionnaires are validated measures of QoL.

IPSS, SHIM, and EPIC-26-bowel questionnaires were given to patients prior to IMRT. Also, the questionnaires were given to patients after IMRT every 3 months during the first year, every 6 months during the second through third years, and annually during the fourth through seventh years of follow-up.

### Intensity Modulated Radiation Therapy

Adjuvant IMRT started within 4 months of radical prostatectomy once any operative side effects had improved. In contrast, salvage IMRT started a median of 25 months postprostatectomy. There was more frequent delivery of tamsulosin hydrochloride in the adjuvant IMRT subgroup. Patients were simulated with an empty rectum using a pelvic CT scan using 3mm cuts. An urethrogram was performed, and 40mL saline mixed with 10mL non-ionic contrast was injected into the bladder at the time of simulation. The European Organization for Research and Treatment of Cancer (EORTC) Radiation Oncology Group guidelines (12) were used to define the post-prostatectomy clinical target volume (CTV) and planning target volume (PTV). These guidelines allow for escalation of the total radiotherapy dose (13). Prescribed total doses were 64.8-72.0Gy using daily 1.8-Gy fractions (2). The median total dose was 70.2Gy using 1.8-Gy daily. The minimum allowable dose delivered to the PTV was 93% of the prescribed dose, and the maximum allowable dose delivered to the PTV was 115% of the prescribed dose. At least 98% of the PTV received ≥95% of the prescribed dose (14). The dosimetric goals for organs at risk were that no more than 25% of bladder or rectal volumes should receive >60Gy.

### Image-Guided Radiation Therapy

Between 2009 and 2013, two of the radiation oncologists who specialize in the treatment of prostate cancer at our center inserted prostate bed fiducials in 45 patients. The other two radiation oncologists who specialize in the treatment of prostate cancer did not use prostate bed fiducials. Three gold fiducial markers were transrectally inserted under local anesthesia at the prior site of the seminal vesicles, right mid lateral prostate, and prostatic apex. The markers made it possible to determine the location of the prostate bed using electronic portal imaging immediately prior to each IMRT treatment. In order to account for inter-fraction organ motion, the patient’s IMRT setup was adjusted each day based on the location of the markers.

#### Androgen Deprivation Therapy

Androgen deprivation therapy (ADT) always consisted of a gonadotropin-releasing hormone agonist. The median duration of a gonadotropin-releasing hormone agonist was 6 months. In some cases, ADT also included an anti-androgen. The median duration of an antiandrogen was one month starting two weeks prior to the gonadotropin-releasing hormone agonist. ADT was given at the discretion of the treating physician to 4/48 (8%) adjuvant IMRT patients and 9/82 (11%) salvage IMRT patients. ADT tended to be used in patients with extraprostatic extension and/or seminal vesicle invasion (pT3 disease). In the adjuvant IMRT subgroup, the median PSA was 0.1ng/mL at the start of radiotherapy in those who did and did not receive ADT. In contrast, in the salvage IMRT subgroup, the median PSA was 0.4ng/mL at the start of radiotherapy in those who did and did not receive ADT. In both the adjuvant and salvage subgroups, the median Gleason score on the prostatectomy specimen was 3+4=7 in those who did and did not receive ADT.

## Phosphodiesterase-5 inhibitors

When used at the discretion of a patient and his physician, a phosphodiesterase-5 inhibitor typically started within the first 3 post-operative months (15). Sildenafil citrate was offered to men with erectile dysfunction, defined as a SHIM score <22, at an initial dose of 50mg two to three times per week. The dose was titrated to 100mg/day if there was no response at 50mg. Alternatively, vardenafil hydrochloride was prescribed at a starting dose of 10mg two to three times/week. This dose was titrated to 20mg up to three times weekly if needed. Men were encouraged to take up to 12 doses per month. Patients were advised to continue taking either medication for at least six doses before considering the drug to be a treatment failure. If one particular oral phosphodiesterase-5 inhibitor failed, the patient was offered an alternative oral agent. Adjuvant IMRT patients elected to use phosphodiesterase-5 inhibitors more often than salvage IMRT patients.

## Definition of Recurrent Disease Post-IMRT

In the adjuvant IMRT group, recurrent disease post-irradiation was defined as a PSA ≥0.2ng/mL with a second confirmatory PSA ≥0.2ng/mL (2). In the salvage IMRT group, progressive disease was defined as a PSA ≥0.2ng/mL above the post-radiotherapy nadir followed by another higher value, a continued rise in PSA despite IMRT, initiation of systemic therapy after IMRT, or clinical progression (16).

## Statistics

Statistical analysis was performed using Statistical Analysis System 9.3 (SAS Institute Inc., Cary, NC, USA). A two-sided t-test was used to calculate the difference in means. A means procedure was used to compute descriptive statistics. A mixed model for repeated measurements was used to compare QoL scores over time amongst subgroups. An α (type I) error <0.05 was considered statistically significant.

## RESULTS

**Figure 1:**
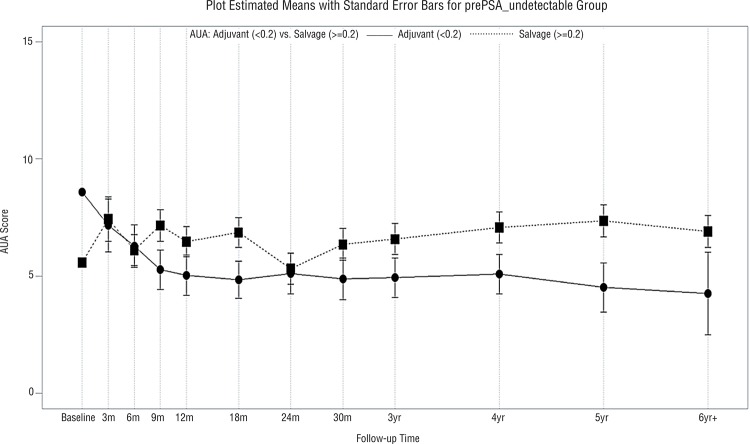
Mean IPSS (urinary QoL) scores with standard error bars in patients who received adjuvant IMRT (─) or salvage IMRT (…) (p=0.03).

**Figure 2:**
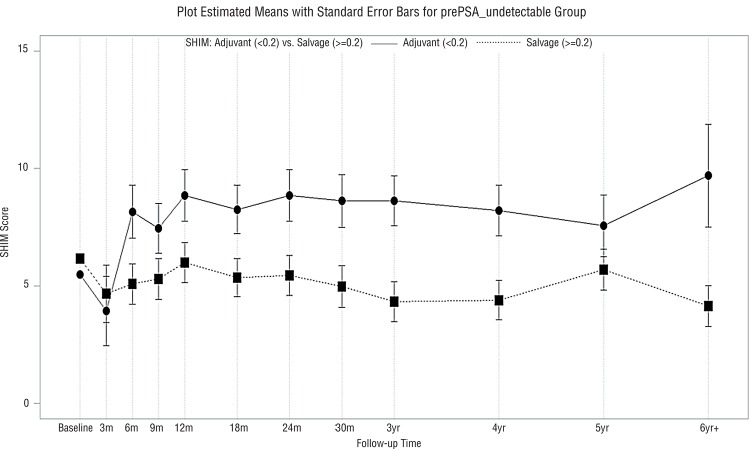
Mean SHIM (sexual QoL) scores with standard error bars in patients who received adjuvant IMRT (─) or salvage IMRT (…) (p=0.002).

**Figure 3:**
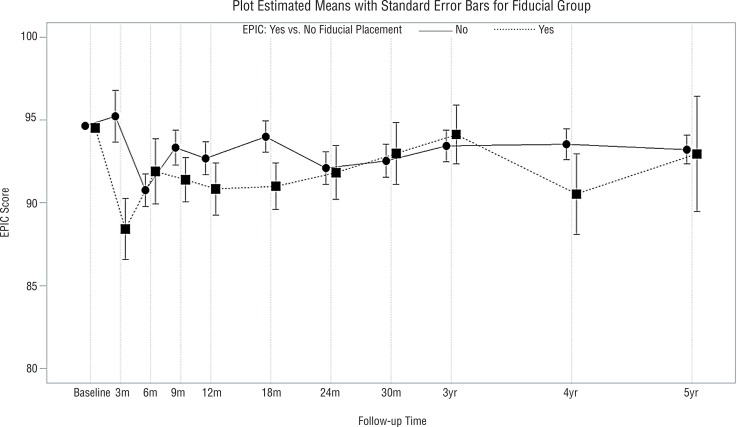
Mean EPIC-26-bowel QoL scores with standard error bars in patients without fiducial markers (─) or with fiducial markers (…) in their prostate bed (p=0.40).

**Figure 4:**
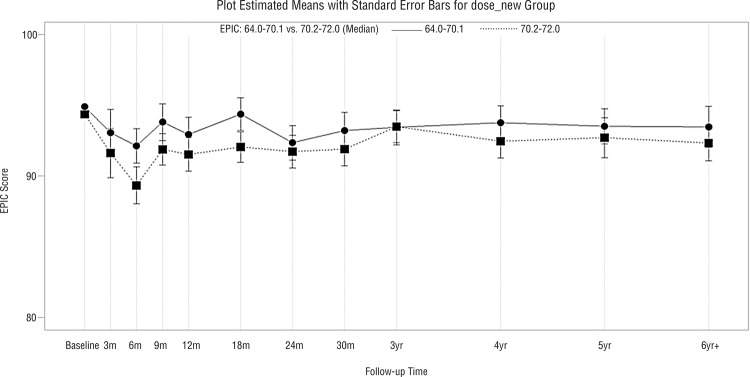
Mean EPIC-26-bowel QoL scores with standard error bars in patients who received total radiotherapy doses of 64.8-68.4Gy (─) or 70.2-72.0Gy (…) (p=0.36).

Median follow-up was 46 months (range, 3-116 months). Characteristics of the adjuvant and salvage radiotherapy patients are presented in Table-1. More patients (58%) in the adjuvant IMRT group had positive surgical margins than patients in the salvage IMRT group (37%) (p=0.02). However, there was no significant difference in the age of the adjuvant IMRT (mean±standard deviation: 61±7 years) versus salvage IMRT (mean±standard deviation: 63±8 years) patients (p=0.16). Similarly, there was no significant difference in the baseline SHIM scores in the adjuvant IMRT (mean±standard deviation: 5±7) versus salvage IMRT (mean±standard deviation: 6±8) patients (p=0.70). Patient compliance with questionnaire completion was 76% over the first 3 years post-irradiation and decreased thereafter. There were significantly better urinary QoL scores in the adjuvant IMRT relative to the salvage IMRT group (p=0.03, Figure-1). Similarly, there were significantly better sexual QoL scores in the adjuvant IMRT relative to salvage IMRT group (p=0.002, Figure-2). Bowel QoL scores did not change significantly after adjuvant or salvage IMRT (p=0.43, Figure-3). The use of prostate bed fiducial markers was not associated with urinary, sexual, or bowel QoL (p=0.39, p=0.49, and p=0.40, respectively). Higher total radiotherapy doses (70.2-72.0Gy versus 64.8-68.4Gy) did not significantly affect urinary, sexual, or bowel QoL (p=0.21, p=0.61, and p=0.36, respectively, Figure-4).

**Table 1:** Characteristics of patients treated with adjuvant and salvage radiotherapy.

Variable	Level	N (%)			P Value

		Total	Adjuvant (<0.2) (n=48)	Salvage (≥0.2) (n=82)	
Gleason Score on Prostatectomy Specimen	6	26 (20%)	6 (13%)	20 (25%)	0.16
	7	91 (70%)	36 (75%)	55 (67%)	.
	8	9 (7%)	3 (6%)	6 (7%)	.
	9	4 (3%)	3 (6%)	1 (1%)	.
	**Total**	**130**	**48 (100%)**	**82 (100%)**	.
Pathologic T Stage	T2a	26 (20%)	7 (15%)	19 (23%)	0.25
	T2b	2 (2%)	0 (0%)	2 (2%)	.
	T2c	61 (47%)	24 (50%)	37 (45%)	.
	T3a	27 (21%)	11 (23%)	16 (20%)	.
	T3b	14 (11%)	6 (13%)	8 (10%)	.
	**Total**	**130**	48 (100%)	**82 (100%)**	.
Pathologic N stage	N0	61 (47%)	22 (46%)	39 (48%)	0.85
	NX	69 (53%)	26 (54%)	43 (52%)	.
	**Total**	**130**	**48 (100%)**	**82 (100%)**	.
M stage	M0	61 (47%)	21 (44%)	40 (49%)	0.58
	MX	69 (53%)	27 (56%)	42 (51%)	.
	**Total**	**130**	**48 (100%)**	**82 (100%)**	.
AJCC stage	I	7 (5%)	1 (2%)	6 (7%)	0.37
	II	83 (64%)	30 (62%)	53 (65%)	.
	III	40 (31%)	17 (35%)	23 (28%)	.
	**Total**	**130**	**48 (100%)**	**82 (100%)**	.
Extraprostatic Extension	No	95 (73%)	33 (69%)	62 (76%)	0.41
	Yes	35 (27%)	15 (31%)	20 (24%)	.
	**Total**	**130**	**48 (100%)**	**82 (100%)**	.
Seminal vesicles invasion	No	116 (89%)	42 (87.5%)	74 (90%)	0.77
	Yes	14 (11%)	6 (12.5%)	8 (10%)	.
	**Total**	**130**	**48 (100%)**	**82 (100%)**	.
Positive Surgical Margins	No	72 (55%)	20 (42%)	52 (63%)	0.02
	Yes	58 (45%)	28 (58%)	30 (37%)	.
	**Total**	**130**	**48 (100%)**	**82 (100%)**	.
Total Dose (Gy) - Median	64.8-68.4	56 (43%)	22 (46%)	34 (41.5%)	0.71
	70.2-72.0	74 (57%)	26 (54%)	48 (58.5%)	.
	**Total**	**130**	**48 (100%)**	**82 (100%)**	.
Androgen Deprivation Therapy	No	117 (90%)	44 (92%)	73 (89%)	0.77
	Yes	13 (10%)	4 (8%)	9 (11%)	.
	**Total**	**130**	**48 (100%)**	**82 (100%)**	.
Fiducials placed in Prostate Bed	No	88 (68%)	31 (65%)	57 (69.5%)	0.6940
	Yes	42 (32%)	17 (35%)	25 (30.5%)	.
	**Total**	**130**	**48 (100%)**	**82 (100%)**	.

## DISCUSSION

Few studies have focused on QoL following post-prostatectomy radiotherapy (17). This study adds to the small body of literature assessing QoL in patients who underwent modern post- prostatectomy radiation using IMRT to the prostate bed (18, 19). Advantages of this study are that modern radiotherapy techniques were used with relatively uniform planning target volumes. Disadvantages are that it is a retrospective study with the resulting potential for selection bias in subgroups. For example, there was an imbalance in the use of Tamsulosin hydrochloride and phosphodiesterase-5 inhibitors. Also, the study has a limited number of patients (n=130). In addition, QoL trajectory after surgery and before adjuvant or salvage IMRT was not examined. Moreover, only 76% of patients completed QoL questionnaires over the first 3 years post-irradiation, with worsening compliance thereafter. Similarly, other groups have reported 36-78% patient compliance rates with questionnaire completion (20, 21).

A key concern of clinicians and patients is that post-prostatectomy radiotherapy will cause deterioration in urinary, sexual, and bowel QoL (17). As a result, more than three quarters of North American prostatectomy patients with either adverse pathological features or an early rise in their postoperative PSA do not undergo post-prostatectomy radiation therapy (17, 22). IMRT is the preferred technique in the United States for post-prostatectomy radiotherapy since it can result in less acute toxicity (17, 23) and better urinary and bowel QoL compared with three-dimensional conformal radiation therapy (3DCRT) (18, 19). Consequently, this QoL report was limited to patients who underwent post-prostatectomy IMRT. In accordance with the findings of others (18, 24) urinary (Figure-1) and bowel (Figure-3) QoL following post-prostatectomy IMRT compare favorably with prior reports on QoL after post-prostatectomy 3DCRT.

In this study, adjuvant IMRT was associated with better urinary QoL than salvage IMRT (p=0.03, Figure-1). This may be related to more frequent use of tamsulosin hydrochloride in the adjuvant IMRT group. Also, this may be due to more rapid recovery of urinary QoL over the first year after radical prostatectomy (25), as is commonly seen in younger patients with few comorbities (26). Adjuvant IMRT started within 4 months of prostatectomy whereas salvage IMRT started a median of 25 months post-prostatectomy. There was no significant difference (p=0.70) in baseline SHIM scores in the adjuvant IMRT versus salvage IMRT groups. However, adjuvant IMRT patients used phosphodiesterase-5 inhibitors more often than salvage IMRT patients. Adjuvant IMRT patients typically started to use phosphodiesterase-5 inhibitors within the first 3 postoperative months. This may help to explain why better sexual QoL was observed in adjuvant IMRT compared with salvage IMRT patients, particular over the first year post-irradiation (Figure-2). The use of early intervention or prophylactic phosphodiesterase-5 inhibitors resulted in improvement in overall sexual function in patients with intact prostate treated with IMRT or brachytherapy (15, 27). Also, it is possible that confounding factors for sexual QoL post-prostatectomy such as prostate size, educational level, or income could have been different between the adjuvant IMRT and salvage IMRT groups (28). Schiffner et al. (29) examined 10 patients who were treated with postoperative radiotherapy and had radio-opaque markers implanted transrectally into the prostate bed using ultrasound guidance. Although the motion of the prostate bed was less than that of an intact prostate, positioning errors exceeded 5mm in many treatment fractions. Therefore, they recommend using daily, image-guided, soft tissue verification with fiducial markers to improve the inter-fraction targeting of the prostate bed. By transrectally placing 3 fiducial markers in the prostate bed under ultrasound guidance, one can improve the accuracy of external beam radiotherapy compared with the use of radical prostatectomy clips (30). However, in the current study, placement of fiducial markers in the prostate bed was not associated with improved urinary, sexual, or bowel QoL (Figure-3).

The National Comprehensive Cancer Network Clinical Practice Guidelines for Prostate Cancer Version 2.2016 recommend adjuvant/salvage post-prostatectomy total radiotherapy doses of 64-72Gy in standard fractionation. In accordance with these guidelines, some groups have recommended higher total radiotherapy doses, i.e., 70-72Gy, in the adjuvant (31, 32) or salvage (33, 34) setting. In the present study, higher total radiotherapy doses did not significantly affect urinary, sexual, or bowel QoL (p=0.21, p=0.61, and p=0.36, respectively, Figure-4). The RAVES (6), Medical Research Council (UK) and National Cancer Institute of Canada Clinical Trials Group Radiotherapy and Androgen Deprivation In Combination After Local Surgery (RADICALS) (35), and French Groupe d’E´tude des Tumeurs Uro-Genitales (GETUG)-17 (36). Phase III studies will clarify whether adjuvant radiotherapy is equivalent to observation with early salvage radiotherapy for those who relapse (5). Moreover, the RADICALS (35), GETUG-16, and radiation Therapy Oncology Group 9601 and 0534 Phase III studies will determine if there is a benefit to adding either 4-6 months or 2 years of ADT to salvage radiotherapy.

In conclusion, there was no significant change in urinary, sexual, and bowel QoL with postprostatectomy IMRT regardless of whether prostate bed fiducial markers or higher total radiotherapy doses were used. QoL with IMRT in the current study compares favorably with prior reports for three-dimensional conformal radiation therapy.
